# Nanostructures for sensors, electronics, energy and environment

**DOI:** 10.3762/bjnano.3.40

**Published:** 2012-05-02

**Authors:** Nunzio Motta

**Affiliations:** 1 School of Chemistry, Physics & Mechanical Engineering, Queensland University of Technology, Brisbane, 4001, Australia

The areas of nanoscale science and technology are rapidly emerging, with a focus on the design, fabrication, and characterization of functional objects on the scale of several nanometers. The implications of the new advances in this field are expected to reach far and wide, spanning a variety of scientific and engineering disciplines. Nanoscale science is growing evermore important on a global scale and is widely seen as playing an integral part in the growth of future world economies.

We are all committed to leaving behind a better world for future generations, and nanotechnology can definitely help to improve our environment in several ways. The daunting energy crisis we are facing could be solved not only by new and improved ways of getting energy directly from the sun, but also by saving power thanks to advancements in electronics and sensors.

New, cheap dye-sensitized and polymer solar cells hold the promise of environmentally friendly and simple production methods, along with mechanical flexibility and low weight, matching the conditions for a widespread deployment of this technology. Their often-criticized scarce efficiency is rapidly growing, thanks to the discovery of new dyes and polymers, which are the fruit of teamwork between chemists, physicists and engineers, all working at the nanoscale.

Cheap sensors based on nanomaterials can make a fundamental contribution to the reduction of greenhouse gas emissions, allowing the creation of large sensor networks to monitor countries and cities, improving our quality of life. Nanowires and nano-platelets of metal oxides are at the forefront of the research to improve sensitivity and reduce the power consumption in gas sensors.

Nanoelectronics is the next step in the electronic roadmap, with many devices currently in production already containing components smaller than 100 nm. Molecules [1–2] and conducting polymers [3] are at the forefront of this research with the goal of reducing component size through the use of cheap and environmentally friendly production methods. This, and the coming steps that will eventually bring the individual circuit element close to the ultimate limit of the atomic level, are expected to deliver better devices with reduced power consumption. In this respect the present Thematic Series is an interesting follow-up to the series “Transport through molecular junctions” published in the Beilstein Journal of Nanotechnology in 2011 [1].

Many of these advances have been possible thanks to the discovery of new aggregation forms of materials at the nanoscale, such as fullerenes, nanotubes, graphene [4] and other carbon structures, or new organic–inorganic mixtures with unexpected properties, discussed also in the series “Organic–inorganic nanosystems” edited by Paul Ziemann [5].

Last but not least, we need to acknowledge that the ability to study these incredible aggregation forms of materials with atomic resolution is mainly due to the developments in scanning probe microscopy [6–7] that have occurred over the last 20 years. The Beilstein Journal of Nanotechnology recently hosted the series “Scanning probe microscopy and related methods” edited by Ernst Meyer [6] and “Noncontact atomic force microscopy” edited by Udo Schwarz [7] to which the interested reader is directed for more information.

Science always holds surprises, and I am delighted to present this Thematic Series, giving a quick glance at the science and application of nanostructures.

Nunzio Motta

Brisbane, April 2012
